# Applicability of a Supported Liquid Membrane in the Enrichment and Determination of Cadmium from Complex Aqueous Samples

**DOI:** 10.3390/membranes8020021

**Published:** 2018-04-23

**Authors:** Núria Pont, Victòria Salvadó, Clàudia Fontàs

**Affiliations:** Department of Chemistry, University of Girona, C/Maria Aurèlia Capmany 69, 17003 Girona, Spain; nuri__pont@hotmail.com (N.P.); victoria.salvado@udg.edu (V.S.)

**Keywords:** solid supported liquid membrane, hollow fibre, Aliquat 336, cadmium, seawater

## Abstract

A supported liquid membrane is developed for the separation of Cd from either high in salinity or acidity aqueous media. The membrane consisted of a durapore (polyvinylidene difluoride) polymeric support impregnated with a 0.5 M Aliquat 336 solution in decaline. The effect of carrier concentration, organic solvent and feed and receiving solutions on the metal permeability is studied. This system allows the effective transport of trace levels of Cd through the formation of CdCl_4_^2−^, which is the predominant species responsible for the extraction process, in both NaCl and HCl solutions. The supported liquid membrane system in a hollow fibre configuration allows the enrichment and separation of trace levels of Cd from spiked seawater samples, facilitating the analytical determination of this toxic metal.

## 1. Introduction

Determination of trace metals in natural waters is still a challenging task due to the low metal concentrations present in these samples and the complexity of the matrices. Despite the availability of highly sensitive analytical tools, the application of separation and selective preconcentration techniques are frequently required prior to the performance of instrumental analysis. Conventional liquid–liquid extraction (LLE) has been the most widely used sample preparation technique to this end. However, LLE methods are time-consuming and use large amounts of high purity organic solvents, which are expensive and potentially toxic.

Liquid membrane (LM) technology is an attractive alternative to traditional LLE for the performance of sample treatment in the determination of trace levels of metal ions that are present in complex matrices [1,2]. A liquid membrane is a barrier interposed between two aqueous phases consisting of an immiscible organic solvent containing a complexing agent (carrier) that is selective towards the target metal [2,3]. One of the aqueous phases, the feed solution, contains the metal ion to be transported whereas the other is the receiving or stripping solution. Therefore, the metal complex formed diffuses across the organic liquid phase. In order to concentrate the metal, the volume of the receiving solution must be much smaller than the volume of the feed solution. Thus, target elements can be separated and concentrated at the same time. Typical liquid membrane configurations [4] are emulsion LM [5], bulk LM [6] and supported LM (SLM) [7,8,9]. In this last case, organic liquid is usually embedded in small pores of a polymer support and held in place by capillary forces. The most important advantages of LM include simultaneous extraction and retro-extraction in a single technological step, possible use of expensive carriers, the high separation factors that can be obtained, the ease of scale-up, the low energy requirements and the low operating costs. The main drawback of SLMs is their instability when they are performing long-term industrial processes [7,8]. However, it is worth noting that the use of ionic liquids (ILs) as a liquid membrane phase, for example, results in the stabilization of the SLMs given the unique properties of ILs, such as their negligible vapour pressure, the possibility of reducing their solubility in the surrounding aqueous phases by the adequate selection of the cation and anion of the IL and their high viscosity that may slow down the displacement of the liquids from the micron pores under pressure [9,10]. Even though stability problems can limit their use in industrial processes, LMs are very useful for improving the sensitivity of analytical methodologies by providing an easy and effective system for matrix clean-up and analyte preconcentration [11,12]. SLMs, in particular, have proved themselves to be an effective sorbent to pre-concentrate metals from both industrial effluents and natural waters in order to facilitate their determination by X-ray fluorescence spectrometry [13,14,15]. Permeation liquid membranes have been successfully applied in metal speciation and bioavailability studies in environmental waters [16,17,18,19].

The flat-sheet geometry, where the planar membrane separates two compartments containing equal volumes of aqueous solutions, is the simplest SLM design. A hollow fibre configuration (HFSLM) is used for preconcentration purposes as it provides a large surface area that allows separation and preconcentration in a single step with high enrichment factors [20,21,22,23]. Both SLM configurations have proved effective for the separation and concentration of a large variety of compounds and metals and many examples can be found in the literature [24,25,26,27,28].

One interesting application of LMs is in environmental analysis, particularly in the determination of heavy metals due to their harmful environmental effects. The US Environmental Protection Agency (EPA) has listed cadmium B1 carcinogen and has limited the concentration in wastewater discharges to a maximum of 2 mg L^−1^. Cd is not biodegradable and so its concentration in the environment steadily increases. Hence, it is of paramount importance to develop a simple methodology to facilitate its determination in complex environmental samples such as seawater.

Although the use of liquid membranes for the transport and separation of Cd have been widely reported in the literature, most of the studies are focused on its recovery from residual solutions and wastewater treatment [29,30]. Thus, few environmental applications of liquid membranes have been reported for the determination of Cd in seawater or other natural waters [31,32,33,34].

Following on from earlier work [35], here we propose to optimize the SLM composition by using anion exchangers as carriers to interact with the Cd anionic complexes formed in chloride media. Parameters affecting both flat-sheet and hollow fibre configurations of the SLM are investigated to allow an efficient separation and enrichment of the metal from complex aqueous samples. Finally, the applicability of the SLM that is developed is investigated for the determination of Cd in seawater samples with the aim of facilitating its monitoring.

## 2. Materials and Methods

### 2.1. Chemicals

Aqueous solutions of Cd and metal mixtures were prepared by dilution of the corresponding stock solution (1000 mg L^−1^, Pure Chemistry, Romil, Cambridge, UK) and the addition of hydrochloric acid (32%) (trace select, Fluka, Bern, Switzerland) or NaCl (Panreac, Barcelona, Spain) to reach the desired chloride concentration. Sodium nitrate (Panreac, Barcelona, Spain) and nitric acid (Fluka, Bern, Switzerland) were used to prepare the stripping solutions. All chemicals were of analytical reagent grade and the solutions were prepared with ultrapure water that was obtained by purification through a MilliQ Plus system (Millipore Iberica S.A., Barcelona, Spain). A synthetic sample containing high amounts of other pollutant metals such as Ni, Cu and Pb in 2 M HCl media was used in the selectivity studies.

The extractants, tricaprylmethylammonium chloride (Aliquat 336) (Fluka, Bern, Switzerland) and tri-*n*-octylamine (TNOA) were used as received. Trilaurylamine chloride (TLAHCl) was prepared by adding hydrochloric acid to the corresponding amine (Merck, Darmstadt, Germany). Cumene (isopropylbenzene) (for synthesis, Merck, Darmstadt, Germany), dihexylether (97%) (Aldrich, Steinheim, Germany), dodecane modified with 4% of dodecanol (Merck, Darmstadt, Germany) and decaline (decahydronaphtalene, *cis* + *trans*) (98%) (Aldrich, Steinheim, Germany) were used as organic solvents.

Spiked seawater samples were prepared by adding the appropriate amount of a Cd stock solution to seawater samples (pH 8.2 and conductivity 62 mS) collected from the Mediterranean Sea (Blanes, Catalonia, Spain). The spiked samples were used without pH adjustment and chloride or ionic strength modification.

### 2.2. Instrumentation

Metal determination in the feed and stripping phases was made by atomic emission spectrometry with an ICP-AES instrument (Varian Liberty RL, Victoria, Australia) whereas Cd determination at low concentration levels in seawater samples was performed by differential pulse anodic stripping voltammetry using a multi-mode electrode 797 VA Computrace (Metrohm, Switzerland). Instrumental parameters and measurement conditions used are described in [15].

### 2.3. SLM in Flat-Sheet Configuration Transport Experiments

Transport experiments in a flat-sheet SLM configuration were carried out by using a two-compartment permeation cell provided with a circular window, where the supported liquid membrane was placed [36]. Durapore film (polyvinylidene difluoride, PVDF) (Merck, Germany) with 125 µm thickness, 0.2 µm pore diameter and 75% porosity was used as the polymeric support [35]. This support was soaked in the carrier solution (normally 0.5 M Aliquat 336 in decaline) for 2 h and then taken out, wiped with a piece of filter paper and then fixed between the two compartments of the SLM cell. The feed and the stripping solutions (200 mL each) were placed in each compartment of the cell. Usually, the feed phase consisted of 10 mg L^−1^ Cd in NaCl or HCl, whereas ultra-pure water was used as a receiving phase [35]. Different aliquots (2 mL) were taken out from both aqueous phases at scheduled time intervals and the Cd concentration was determined by ICP-AES.

All experiments were carried out at ambient temperature (22 ± 1 °C).

The permeability coefficient, *P* (cm min^−1^) was calculated as:

(1) $$p=-\frac{d\left[Cd\left(II\right)\right]}{dt}\cdot \frac{V}{A}\cdot {\frac{1}{\left[Cd\left(II\right)\right]}}_{f,0}$$

where *A* (cm^2^) is the effective membrane area and *V* (cm^3^) the volume of the aqueous feed solution, *t* (s) the time and [*Cd*(*II*)]*_f_*_,0_ the initial cadmium concentration in the feed solution.

The integration of Equation (1), assuming that permeability is independent of time, resulted in the following equation:

(2) $$\ln\frac{{\left[Cd\left(II\right)\right]}_{f,t}}{{\left[Cd\left(II\right)\right]}_{f,0}}=-\frac{A}{V}P\cdot t$$

The linear representation of ln[*Cd*(*II*)]*_f_*_,*t*_/[*Cd*(*II*)]*_f_*_,0_ versus time allows the calculation of the permeability coefficient, *P*, from the slope of the straight line.

### 2.4. Hollow Fibre Supported Liquid Membrane (HFSLM) Experiments

**Figure 1:**
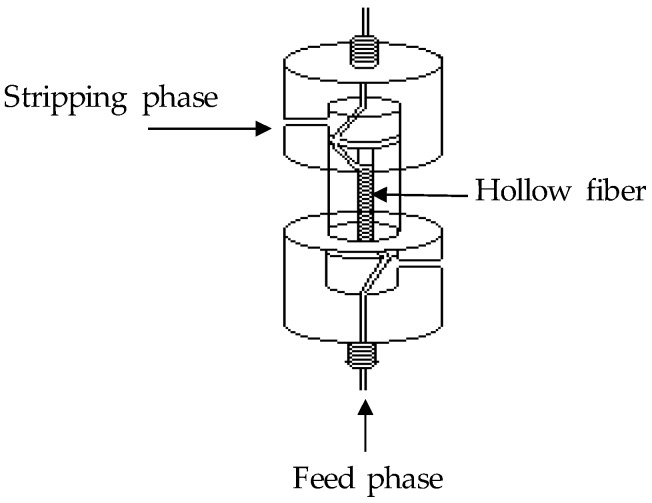
Scheme of the module used in the hollow fibre supported liquid membrane (HFSLM) experiments.

Experiments for the simultaneous transport and enrichment of Cd were carried out using a HF module, which contained one coiled hollow fibre (see Figure 1). In this case, hydrophobic polypropylene hollow fibres, from Azko (Enka, E. G., Frankfurt, Germany) were used as the liquid membrane support with the following characteristics: inner diameter = 0.3 mm, outer diameter = 0.5 mm, pore size = 0.2 µm, porosity = 75%, length = 57 cm which results in an effective inner membrane area of 5.4 cm^2^.

The HFSLM was prepared by impregnation of the tubular microporous with a solution of Aliquat 336 in decaline (0.5 M or 0.05 M) by slow passing it through the lumen of the hollow fibre. Usually, 100 mL of the feed and 5 mL of the stripping solutions were continuously recirculated, using a Gilson peristaltic pump (Pacisa, Barcelona, Spain) at a flow rate of 1 mL min^−1^, by the lumen and the shell-side of the fibre, respectively. The experimental set-up is described in [36]. Each experiment was conducted over a period of about 24 h.

Moreover, the SLM system can be also evaluated in terms of metal extraction. The percentage of Cd extraction, *E*(%), was calculated by Equation (3):

(3) $$E(\%)=\frac{{\left[Cd\right]}_{feed_{\left(0\right)}}-{\left[Cd\right]}_{feed_{\left(t\right)}}}{{\left[Cd\right]}_{feed_{\left(0\right)}}}\times 100$$

where [*Cd*]*_feed_*_(0)_ is the initial Cd concentration in the water sample whereas [*Cd*]*_feed_*_(*t*)_ is the metal concentration in the source solution at the end of the experiment (24 h).

Cd transport efficiency (TE) in both SLM configurations, flat-sheet and HF, was determined by Equation (4):

(4) $$TE(\%)=\frac{{\left[Cd\right]}_{strip_{\left(t\right)}}}{{\left[Cd\right]}_{feed_{\left(0\right)}}}\;\frac{1}{Vr}\times 100$$

where [*Cd*]*_strip_*_(*t*)_ denotes the Cd concentration in the stripping compartment at the end of the contact time. *Vr* is the ratio between the volumes of the feed and stripping solutions. For flat-sheet experiments *Vr* = 1, while for HFLSM *Vr* = 20.

## 3. Results and Discussion

The design of a membrane system requires the selection of the chemical conditions and physical parameters that result in a highly efficient transport of the target metal. Taking into account our preliminary results [35], Durapore was used as the polymeric support in the present study and ultra-pure water was fixed as the stripping phase.

### 3.1. Composition of the Liquid Membrane

As cadmium forms anionic complexes in hydrochloric acid during the leaching of ores and secondary materials and given that similar species can be found in seawater, we investigated the composition of a liquid membrane that is able to extract Cd chlorocomplexes. Among other reagents, these anionic complexes may be extracted by protonated amines or quaternary ammonium cations. It is well known that both tertiary amines as well as the quaternary ammonium salt Aliquat 336 effectively extract Cd from highly acidic solutions [6,37,38]. Amines require a sufficiently low pH to be protonated. He at al. [6] describe the extraction of Cd(II) ions in HCl solution with TNOA and TLA in solvent extraction experiments. Both tertiary amines accept one proton to form a neutral species and thus Cd(II) can be extracted as (R_3_NH)_2_CdCl_4_. 

**Figure 2:**
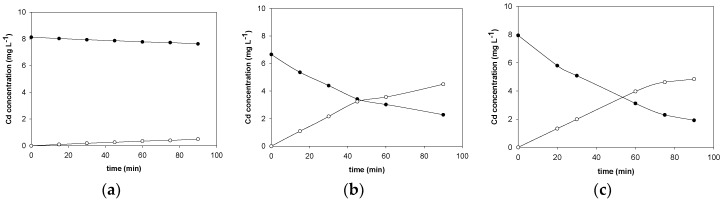
Transient concentration curves in Cd(II) transport experiments involving Trilaurylamine chloride (TLAHCl) (**a**); Tri-*n*-octylamine TNOA (**b**) and Aliquat 336 (**c**). Closed circles: feed phase; open circles: stripping phase.

These two amines and Aliquat 336 were tested as carriers in this study to transport Cd from a NaCl media instead of HCl. For both Aliquat 336 and TLAHCl the feed phase consisted of 10 mg L^−1^ in 2 M NaCl whereas in the case of TNOA the feed composition was 1.9 M NaCl and 0.1 M HCl, to ensure the protonation of the amine. The carrier concentration was set at 0.5 M and due to the poor solubility of TLAHCl in aliphatic solvents, cumene was used as the organic solvent. A comparison of the transport efficiency of these three carriers (Figure 2) showed that among the two amines tested, TNOA allowed an efficient transport of Cd, whereas in the case of TLAHCl, transport was slow. On the other hand, Aliquat 336 permitted fast and effective Cd transport. This last carrier presents the advantage that it can be used in both acidic or neutral solutions since it is able to exchange the chloride initially present in its formulation by the anionic complex of Cd without a previous protonation of the N group. Thus, further experiments were performed using Aliquat 336 as a carrier.

The selection of an appropriate organic solvent is an important parameter in order to obtain stable flat-sheet SLMs. Moreover, the physico-chemical properties of the diluents affect the stability of the polymeric support and the solubility of the complex formed in the organic phase. Thus, besides the aromatic solvent tested (cumene), three aliphatic solvents such as decaline [39], dodecane modified with dodecanol [23,25] or dihexylether, that have been proved to be effective in SLM systems, were also investigated. Similar permeability values were obtained with the aliphatic diluents while cumene gave the highest value (Table 1). Finally, decaline was the selected solvent as aromatic solvents such as cumene can damage the polymeric support due to the swelling of the polymer, leading to an increase of the pore size and causing the loss of the liquid membrane [36].

**Figure 3:**
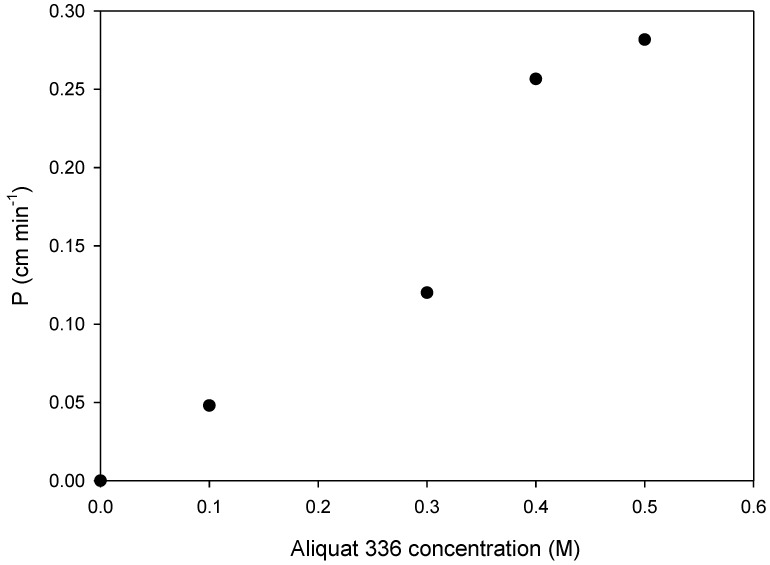
Effect of Aliquat 336 concentration in decaline on Cd permeability. Feed phase: 10 mg L^−1^ Cd in 2 M NaCl.

The effect of carrier concentration on metal transport was also studied. Figure 3 shows the permeability values for Cd(II) through a SLM impregnated with different solutions ranging from 0 to 0.5 M Aliquat 336 in decaline. It can be observed that the absence of a carrier results in a null transport of Cd and that *p* values increase as the carrier content increases. It can therefore be considered that in the transport process permeability is controlled only by membrane diffusion [40]. Further experiments were performed fixing a carrier concentration of 0.5 M, which ensured the maximum transport efficiency.

### 3.2. Effect of Chloride Concentration and pH of the Feed Phase

**Figure 4:**
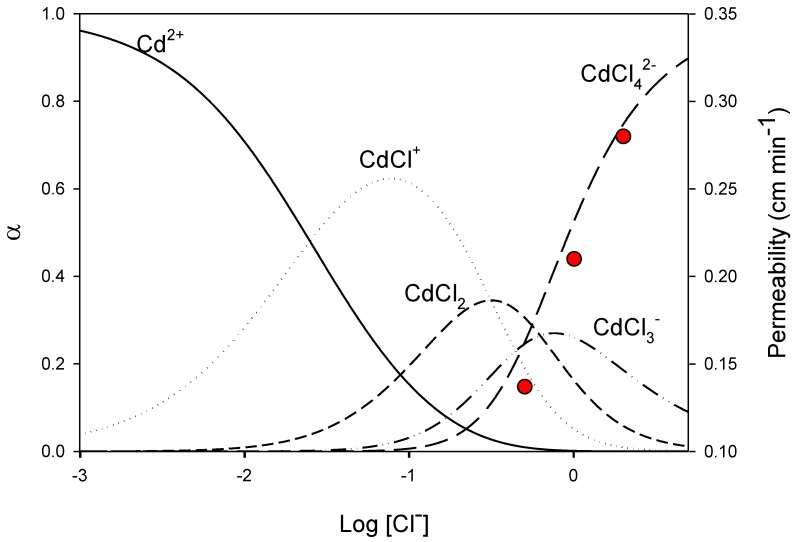
Variation of SLM permeability to Cd (closed circles) versus log [Cl^−^] together with the speciation diagram of Cd (10 mg L^−1^).

In order to study the influence of chloride concentration in the feed phase on cadmium transport, a set of experiments was carried out working with 10 mg L^−1^ Cd at various chloride concentrations provided by NaCl. In Figure 4, cadmium permeability values are depicted together with the different Cd species present against chloride concentration. As can be seen, *P* values increase in line with increasing chloride concentrations, which in turn is related with the predominance of the CdCl_4_^2−^ species. These results seem to indicate that this chlorocomplex is responsible for the transport process, unlike what is postulated in polymer inclusion membranes (PIM). In this last case, the species CdCl_3_^−^ is assumed to play the most important role in the extraction process [37]. This difference can be associated with the different viscosity of the SLM and the PIM, since the lower viscosity of the SLM can facilitate the formation of the species (R_3_R’N)_2_CdCl_4_ (with R_3_R’N being the cationic part of Aliquat 336) in the organic phase.

Moreover, to investigate whether the pH (from HCl) of the feed phase affected Cd transport, an experiment using 10 mg L^−1^ Cd in 2 M HCl was run. The permeability value in this case was 0.260 cm min^−1^, which is very similar to that obtained in NaCl (0.282 cm min^−1^) indicating that the SLM system is not affected by the high acidity of the feed solution containing HCl. 

### 3.3. Application of the SLM to Determine Cd in Complex Liquid Samples

In order to test the suitability of the separation method for Cd enrichment, some metallic species usually present in contaminated liquid environmental samples and in industrial effluents were studied as potential interferents for Cd transport. For such an approach, a synthetic water sample containing about 0.5 mg L^−1^ of Cd with about 10 mg L^−1^ of Ni, Cu and Pb in 2 M HCl was used as a feed phase. Results are presented in Table 2, and, as can be seen, Cd was quantitatively transported in only 2 h of experiment. At this time, no other metal was significantly present in the stripping phase, showing the high selectivity of the SLM towards Cd even in the presence of large amounts of other metals. However, when the SLM was run for 24 h a small amount of Cu (0.5 mg L^−1^) and 2 mg L^−1^ Pb were transported, which is of interest if the removal of these metals is required.

**Figure 5:**
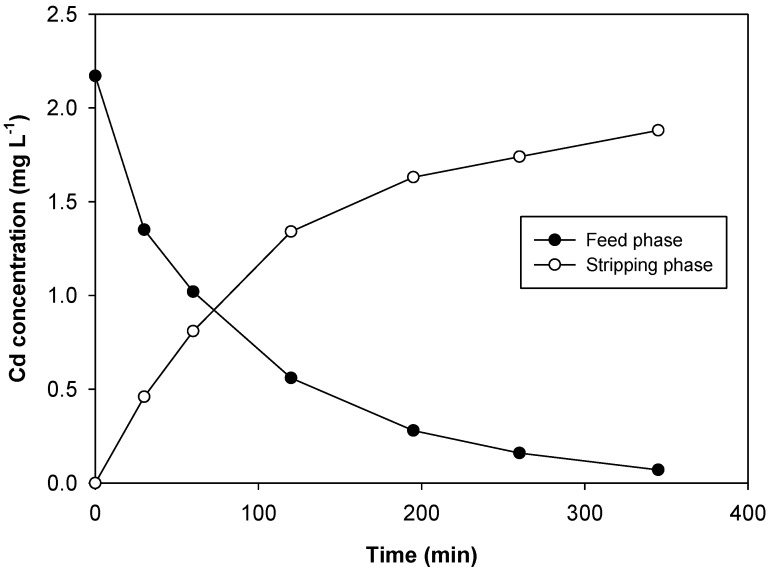
Transient concentration curves in Cd transport experiments in spiked seawater sample.

The effect of a complex matrix such as seawater on Cd transport was also investigated. As stated in the introduction, the determination of trace metals in seawater is difficult due to several factors, most importantly because of the very low concentrations and the high salt content of the sample matrix. Moreover, since Aliquat 336 is able to interact with all the anions present in the sample, the transport of Cd can be affected by several interferences. As can be seen in Figure 5, despite the complexity of the matrix and the presence of large amounts of other anions, Cd was effectively transported to the stripping phase, with a similar P value to that obtained in a control experiment done using 2 mg L^−1^ Cd in 1 M NaCl (*P* = 0.275 cm min^−1^ in seawater and *P* = 0.258 cm min^−1^ in NaCl) as a feed phase. Taking into account that Cd is released in an ultrapure water phase, it greatly facilitates the determination of the metal. 

### 3.4. Preconcentration of Cadmium with a Hollow Fibre System

**Figure 6:**
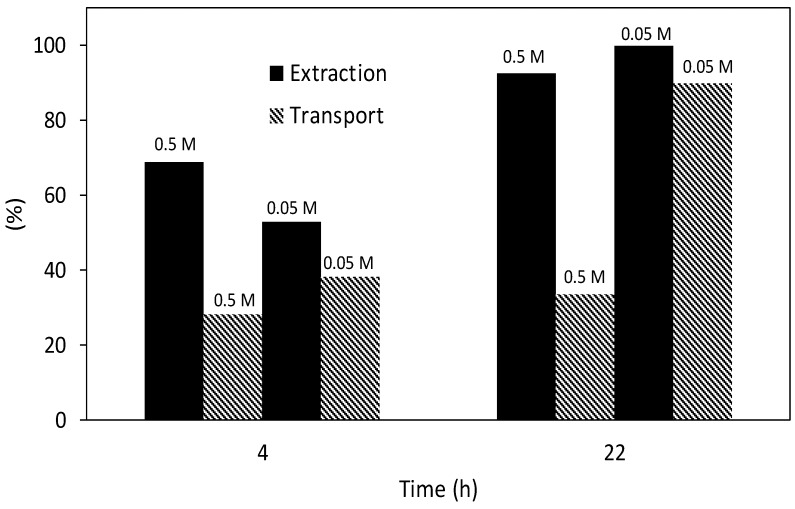
Influence of Aliquat 336 concentration on Cd(II) extraction and transport efficiency using a HFSLM. Feed solution: 2 mg L^−1^ Cd in 1 M NaCl.

In order to test the feasibility of the proposed SLM system for the preconcentration of Cd in high salinity samples, some experiments were performed in a hollow fibre configuration using 2 mg L^−1^ in 1 M NaCl as a feed phase. When the fibre was impregnated with an 0.5 M Aliquat 336 in decaline solution the extraction was highly effective after 22 h of experiment, as can be seen in Figure 6.

However, the transport efficiency was low and, hence, most Cd was retained in the SLM. Thus, a lower concentration of Aliquat 336 was tested and results are also shown in the same figure. As can be seen, an 0.05 M concentration of carrier allowed the quantitative transport of Cd in 22 h.

These better results for the most diluted Aliquat 336 solution can be explained in terms of the viscosity of the organic solution that produces increased membrane resistance at high carrier concentrations. This composition was therefore chosen for the application studies.

Finally, the HFSLM system developed was applied to the preconcentration of cadmium in spiked seawater samples at the level of 20 µg L^−^^1^. Results are presented in Table 3. It is worth mentioning that the samples were used without any previous treatment of pH modification or filtration. The initial metal content was analysed by AV whereas the metal preconcentrated in the stripping phase could be analysed by ICP-OES.

## 4. Conclusions

Cadmium can be effectively separated and preconcentrated from solutions with both high content of NaCl or HCl through a solid supported liquid membrane containing 0.5 M Aliquat 336 in decaline. Even though Aliquat 336 is a non-selective extractant, the possible transport of other anions does not affect the transport of Cd, even at low level concentrations or in a complex matrix such as seawater. The viscosity of the SLM has been shown to be a critical parameter when using a hollow fibre configuration, for which a lower concentration of carrier gives greater transport efficiency. The HFSLM that is developed here has allowed the enrichment of the metal present at ultra-trace levels from a seawater sample to an ultrapure water sample and thus, facilitates the detection of cadmium in such a complex matrix. Due to the easy preparation and high efficiency of the SLM, it can be viewed as a good alternative for the purpose of monitoring Cd in seawater.

## Figures and Tables

**Table 1:** Effect of the organic diluent on Cd permeability. Feed phase: 10 mg L^−1^ Cd in 2 M NaCl; Aliquat 336 concentration: 0.5 M.

Organic Solvent	*P* (cm min^−1^)
Decaline	0.282
Dihexylether	0.263
Dodecane (4% dodecanol)	0.252
Cumene	0.356

**Table 2:** Selectivity studies of the SLM system. Feed phase: metals in 2 M HCl; SLM: 0.5 M Aliquat 336 in decaline.

Metal	(Metal) Feed, 0 (mg L^−1^)	TE (%) (2 h/24 h)
Cd	0.486	100/100
Cu	9.622	0/2
Ni	9.209	0/0
Pb	9.169	0/21

**Table 3:** Cd transport efficiency from spiked seawater samples using the designed HFSLM system.

Sample	Cd Added (mg L^−1^)	Cd Stripping (24 h) (mg L^−1^)	TE (%) (24 h)
Seawater	0.026	0.439	84.4
	0.019	0.377	99.2
